# Towards the inclusion of managed macroalgal ecosystems in the IPCC greenhouse gas inventory

**DOI:** 10.1093/nsr/nwaf391

**Published:** 2025-09-16

**Authors:** Xiuzhen Li

**Affiliations:** State Key Laboratory of Estuarine and Coastal Research, East China Normal University, China

Macroalgae ecosystems have very high productivity, with ∼0.5 and 1 kg C/m^2^/a for dominant wild species and seaweed aquaculture, respectively [1]. However, the instant blooming and massive degradation of certain wild macroalgae, such as *Ulva* spp. (green tides) and *Sargassum* spp. (golden tides) often cause temporal environmental hazards such as hypoxia or ocean acidification [2,3]. While considerable research has focused on bloom dynamics, dispersal, control and adaptation strategies [2,4], the fate of sunken macroalgal biomass and its potential as a carbon sink have received less attention. The large-scale sinking of macroalgae onto continental shelves and deep-sea environments represents a significant yet under-explored pathway for carbon sequestration [5]. The incomplete understanding of underlying mechanisms has hindered the inclusion of these ecosystems in carbon trading frameworks [6]. Investigations on these issues are essential for the management of macroalgae as a potential nature-based solution for climate mitigation [1,7].

A recent study [8] sheds new light on the fate of the sunken macroalgal biomass. Through a 2-year microbial degradation test of sunken *Ulva prolifera*, the authors demonstrated that ∼38% of the initial macroalgal biomass carbon was converted into long-term sequestration forms: recalcitrant organic carbon (28%) and stable dissolved inorganic bicarbonate ions (10%). This transformation was facilitated by the ‘microbial carbon pump’ and ‘microbially driven alkalinity pump’. Given the coverage area of green tides spanning from hundreds to thousands of square kilometers in the Chinese Yellow Sea every year in the last decade, if 5–10 million tons of fresh *U. prolifera* sink into the seafloor annually, they may permanently remove up to 3 × 10^5^–6 × 10^5^ tons of CO_2_ from the atmosphere, according to the results of this study. These findings underscore the potential of managed sinking strategies to enhance carbon storage while mitigating negative ecological impacts. This study also sheds inspiring light on new technologies to control macroalgae blooming and enhance carbon sequestration, such as using natural minerals to stabilize organic carbon and to alleviate the negative effects during early-stage degradation of sinking macroalgae [3].

In addition to wild macroalgae, artificially cultivated macroalgae such as kelp also produce a large amount of shedding material and exudate to the ambient water during their growth and harvesting processes, ultimately forming long-term carbon sinks [7]. According to Feng *et al.* [9], one kelp can provide ∼52 grams of recalcitrant carbon during its life cycle, equivalent to its harvested biomass carbon, highlighting the dual role of seaweed farming in biomass production and carbon export.

More and more countries and organizations are encouraging the inclusion of managed macroalgae ecosystems into the IPCC Greenhouse Gas Inventory [10]. With more evidence presented like Li *et al.* [8], there will be enough confidence to incorporate this important type of ecosystem into climate mitigation accounting. Future research should focus on field validation, scaling mechanisms, and the integration of microbe-macroalgae interactions into ecosystem models. Moreover, policy frameworks should also be developed to quantify, verify, and promote macroalgal carbon sequestration across natural and farmed systems.

In conclusion, the study by Li *et al.* [8] represents a critical step towards understanding and leveraging macroalgal ecosystems for climate mitigation. It provides a scientific basis for developing targeted technologies and policies that transform macroalgal blooms from ecological threats into sustainable carbon sinks.
